# Carmi C. Franklin, New York City

**Published:** 1862-08

**Authors:** J. S. Latimer


AT a meeting of the New York City Dental Association, held at the Cooper Institute, on the evening of June 18th, 1862, Dr. Geo. H. Perine presented the following preamble and resolutions, which were unanimously adopted:

WHEREAS, It hath pleased Almighty God, in his wise providence, by a sudden death in early manhood, to remove from the scene of his earthly labors Carmi C., eldest son of our friend and co-laborer, Dr. B. W. Franklin, therefore

*Resolved*, That as members of the profession, while we deplore his loss, we gratefully cherish in our hearts the memory of our late friend and professional brother, whose pursuits, with his talents and attainments, would have secured him an honorable position.

*Resolved*, That we tender to his bereaved parents, in this, the hour of their deepest affliction, our heartfelt sympathy.

*Resolved*, That copies of these resolutions be forwarded by our Recording Secretary to the family of the deceased, and to the Dental Cosmos and Dental Register; furthermore, that they be entered on the Minutes. J. S. Latimer, *Sec'y.*

